# Functional Characterization of *MaSPL8* Reveals Its Different Roles in Biotic and Abiotic Stress Responses in Mulberry

**DOI:** 10.3390/plants14060950

**Published:** 2025-03-18

**Authors:** Longyan Zheng, Wenhao Zhang, Liuqing Wei, Mengqi Li, Li Liu

**Affiliations:** 1 Jiangsu Key Laboratory of Sericultural Biology and Biotechnology, School of Biotechnology, Jiangsu University of Science and Technology, Zhenjiang 212100, China; 2 Key Laboratory of Silkworm and Mulberry Genetic Improvement, Ministry of Agriculture and Rural Affairs, Sericultural Research Institute, Chinese Academy of Agricultural Sciences, Zhenjiang 212100, China

**Keywords:** *MaSPL8*, mulberry, biotic stress, abiotic stress, phytohormone, VIGS

## Abstract

The Squamosa promoter-binding protein-like (SPL) family proteins plays pivotal roles in plant development and stress adaptation. In this study, we functionally characterized *MaSPL8* in mulberry (*Morus alba*) and investigated its regulatory roles in biotic and abiotic stress responses. *MaSPL8* encodes a 364-amino acid protein with a conserved SBP domain and lacks *miR156/157* binding sites. Phylogenetic analysis confirmed its orthology to *Arabidopsis* AtSPL8, albeit with functional divergence. Downregulation of *MaSPL8* via virus-induced gene silencing (VIGS) resulted in more susceptibility to *Ciboria shiraiana* infection, but significantly enhanced resistance to drought and salt stress, as evidenced by reduced oxidative damage, elevated proline accumulation, and increased antioxidant enzyme activities. Transcriptomic profiling of *MaSPL8*-silenced plants revealed enrichment of differentially expressed genes (DEGs) in brassinosteroid biosynthesis, jasmonic acid metabolism, and oxidative stress responses, suggesting hormone signaling interplay. Furthermore, bioinformatic predictions identified *miR5658* and *miR4221* as potential post-transcriptional regulators of *MaSPL8*. This study highlights *MaSPL8* as a negative regulator of abiotic stress tolerance and positive regulator of biotic (*C. shiraiana*) stress tolerance in mulberry and provides insights into its integration with phytohormone pathways. Our findings underscore the evolutionary plasticity of *SPL8* genes and propose *MaSPL8* as a target for enhancing mulberry’s resilience in challenging environments.

## 1. Introduction

Squamosa promoter-binding protein-like (SPL) proteins constitute a diverse plant-specific transcription factor family and are recognized as key regulators of plant growth and development [1]. These proteins are defined by a highly conserved SBP domain, composed of 78 amino acid residues that form two structural motifs: a bipartite nuclear localization signal (NLS) and a zinc finger motif containing two Zn^2+^-binding sites (Cys-Cys-His-Cys and Cys-Cys-Cys-His) [2,3]. The SBP domain facilitates dual functions: nuclear import via the NLS and sequence-specific DNA binding to the GTAC core motif in target promoters [4,5].

*SPL* genes are found in all green plants, including green algae, mosses, ferns, gymnosperms, and angiosperms [2,5]. The first two *SPL* genes, *AmSBP1* and *AmSBP2*, were found in *Antirrhinum majus* and proved to be involved in the control of early flower development by binding to the promoter of the floral meristem identity gene *SQUAMOSA* (*SQUA*) [6]. The *SPL* gene family has been identified in various plant species including *Arabidopsis thaliana* (At), *Oryza sativa* (Os), *Zea may* (Zm), *Citrus Clementina* (Cc), *Populus trichocarpa* (Ptr), and *Triticum aestivum* (Ta) [2,7,8,9,10,11,12,13]. Phylogenetic analyses classify *SPL* genes into five to ten clades, reflecting lineage-specific diversification during evolution [2,8,12,14]. Functionally, SPLs are divided into two types based on the presence or absence of *miR156/157* binding sites [15]. *miR156/157* are core regulators targeting most SPLs including *Arabidopsis AtSPL3/4/5/9/10* and rice *OsSPL14*, controlling phase transition, tillering, root development, and panicle development [15,16,17], while non-canonical regulators such as maize SPLs may interact with *miR529/535*. Bioinformatic analyses suggest that SPL in maize might have binding sites for *miR529* or *miR535* [18,19]. *miR529* is evolutionarily related to the *miR156* family and modulates drought responses in maize [19,20]. Beyond plant development, *SPL* genes such as peanut (*Arachis hypogaea* L.) *AhSPL5* and wheat *TaSPL6* also affected plant resistance to abiotic stresses such as drought and salt stresses [21,22].

SPL8 belongs to the Group III clade based on the classification in *Arabidopsis*, which is conserved in both monocots and dicots but shows functional divergence [10,23]. Unlike most SPLs, *SPL8* lacks miR156/157 binding sites but is potentially regulated by *miR529/535* in maize [18]. In *A. thaliana*, *AtSPL8* regulates reproductive development, including megasporogenesis, trichome patterning, and male fertility [15,24,25,26]. Functional parallels and contrasts exist across species. *Medicago sativa MsSPL8* regulated phase transition and inflorescence development via *SEPALLATA3* (*SEP3*) and *MADS32* activation and *MsSPL8* suppression improves biomass yield and stress tolerance [27]. Similarly, *Codonopsis pilosula CpSPL8* negatively regulates salt resistance by repressing the *CpSOS2* pathway [28]. Notably, *SPL8* intersects with phytohormone signaling to balance development and stress adaptation. For example, AtSPL8 synergizes with gibberellin signaling during anther development and interacts with brassinosteroid-associated BIM1 to regulate fertility [26,29]. Intriguingly, altering the expression levels of the *SPL8* gene in *Medicago truncatula* has been shown to influence the gibberellic acid (GA) content within the plant. GA levels were significantly elevated in *Mtspl8* mutant plants, whereas they were reduced in the *MtSPL8* overexpression plants, suggesting crosstalk between *SPL8* and GA signaling [27,30].

Mulberry (*Morus* spp.) is a plant of great economic and ecological importance, serving as a cornerstone of sericulture. Its fruits are rich in bioactive compounds, making them valuable in the food and pharmaceutical industries [31,32]. Additionally, mulberry plays a vital role in environmental protection and ecological restoration and is commonly used for windbreaks, soil conservation, and heavy metal remediation [33,34]. Understanding its functional genes is crucial for improving stress resistance, optimizing cultivation strategies, and advancing industrial applications. Preliminary genome-wide analyses have identified multiple SPL homologs in *Morus notabilis*, but functional studies are scarce [35]. Most *SPL* genes in mulberry remain underexplored. Our previous study identified *MaSPL8* as a sclerotiniose-responsive gene (SRG) during *C. shiraiana* infection [36]. Here, we further demonstrate that *MaSPL8* acts as a negative regulator of resistance to biotic (*C. shiraiana*) and abiotic (drought, salinity) stresses. Additionally, we reveal its potential interplay with brassinosteroid and jasmonic acid signaling. Given mulberry’s ecological resilience, deciphering the roles of SPL8 in this species could unlock strategies for enhancing stress tolerance and biomass production.

## 2. Results

### 2.1. Molecular Cloning and Characterization of MaSPL8 in Mulberry

**Figure 1:**
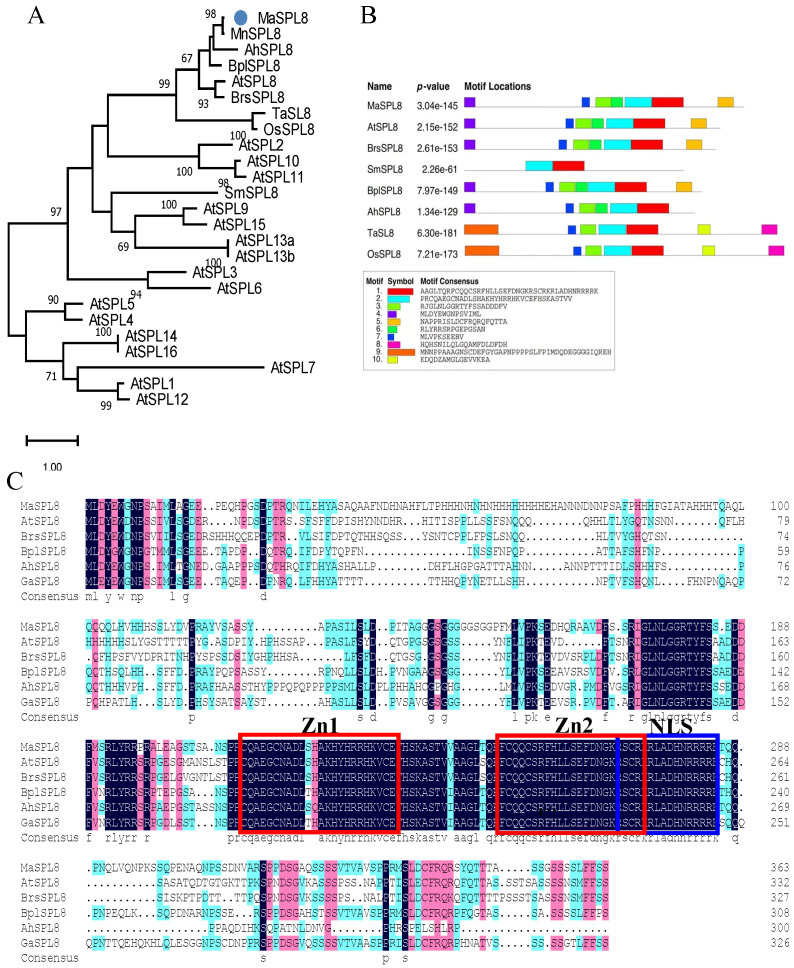
Characterization of MaSPL8 protein sequence. (**A**) Phylogenetic analysis of SPLs from *Morus alba* and other plant species. SPLs from *A. thaliana* were extracted from the Arabidopsis Information Resource (TAIR). Accession numbers for SPL8 for different plant species are as follows: *Brassica rapa* BrsSPL8, XP_009119750; *Betula platyphylla* BplSPL8, AXB72472; *Arachis hypogaea* AhSPL8, XP_025640943; *Gossypium arboreum* GaSPL8, XP_017641940; *Triticum aestivum* TaSPL8, KAF7018911; *Oryza sativa* OsSPL8, NP_001406835; *Morus notabilis* MnSPL8, XP_010089258; *Salvia miltiorrhiza* SmSPL8, AIE89797. (**B**) Conserved motifs of SPL8 from different plant species. (**C**) Alignment of SPL8s from different plant species. Zn1 and Zn2 binding motifs are indicated by red boxes and NLS is indicated by a blue box.

*MaSPL8* was cloned from *Morus alba* and further bioinformatic characterization of *MaSPL8* was performed. The coding sequence (CDS) of *MaSPL8* spans 1095 bp, encoding a 364-amino acid protein. Phylogenetic analysis demonstrated that MaSPL8 is the ortholog of *A*. *thaliana* AtSPL8 (Figure 1A). Conserved motif analysis revealed that MaSPL8 shares identical motif composition and arrangement with SPL8 homologs from other dicots (Figure 1B). Sequence alignment further confirmed the presence of an SBP domain in MaSPL8 and reference SPL8 proteins, with conserved Zn1/Zn2 domains and nuclear localization signals (NLSs) marked (Figure 1C). MaSPL8 lacks *miR156/157* regulatory motifs, consistent with the functional divergence from miR156-regulated clades I/II. Bioinformatic prediction identified two novel miRNAs, *miR5658* and *miR4221*, as potential post-transcriptional regulators of *MaSPL8* (Table 1).

### 2.2. MaSPL8 Mediates Responses to Biotic and Abiotic Stresses

**Figure 2:**
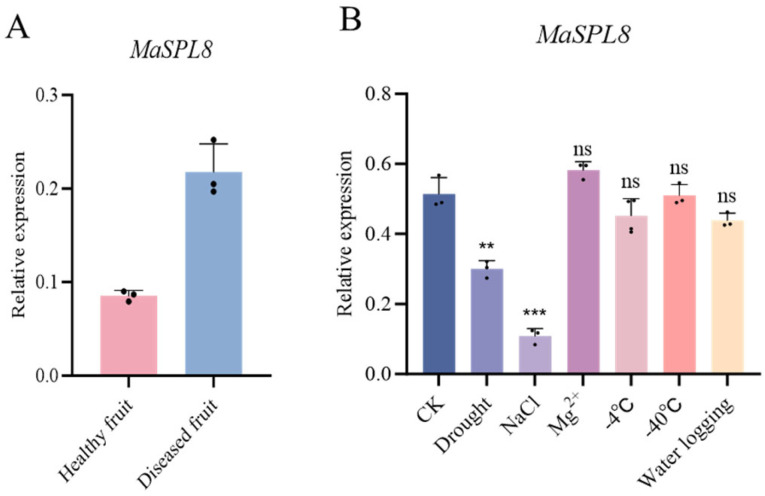
Expression profile of *MaSPL8* in response to various stresses in mulberry. (**A**) Expression levels of *MaSPL8* in response to sclerotiniose; (**B**) expression levels of *MaSPL8* in response to different abiotic stresses. Data are presented as means ± SD of at least three biological replicates. The significance was marked using ** (0.001 < *p* < 0.01), *** (*p* < 0.001), ns (non-significant).

Our previous transcriptomic study identified *MaSPL8* as a sclerotiniose-responsive gene (SRG) [36]. Upon *C. shiraiana* infection, *MaSPL8* expression was significantly upregulated in diseased fruits (Figure 2A). In contrast, exposure to drought or salt stress markedly suppressed *MaSPL8* expression (Figure 2B). It is likely that *MaSPL8* plays roles in responses to both biotic and abiotic stresses.

### 2.3. VIGS-Mediated MaSPL8 Silencing Impairs Growth and Enhances Pathogen Resistance

**Figure 3:**
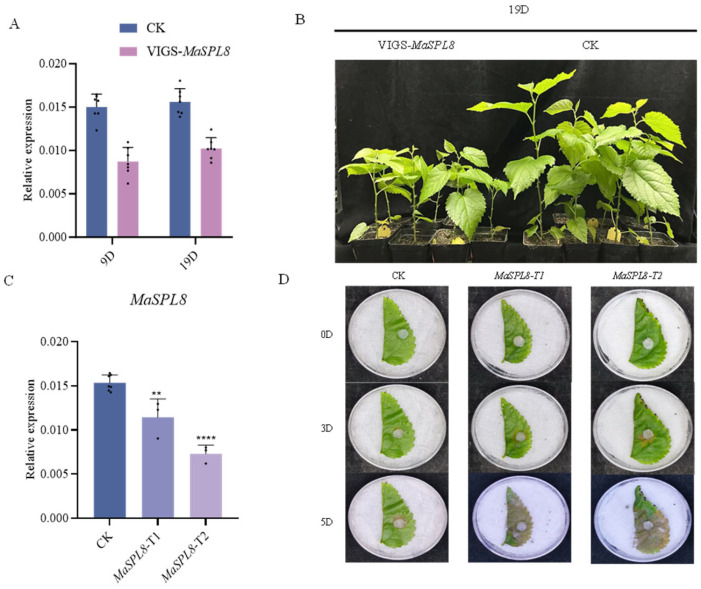
Downregulation of *MaSPL8* by VIGS impairs growth and enhances pathogen resistance in mulberry. (**A**) qRT-PCR (quantitative real-time PCR) detection of *MaSPL8* expression levels in VIGS-treated mulberry plants 9 days and 19 days post-treatment. (**B**) Growth conditions of VIGS-treated mulberry and controls 19 days post-treatment. (**C**) qRT-PCR detection of MaSPL8 expression levels in VIGS-treated mulberry and controls used for *C. shiraiana* infection. (**D**) Observation of cell death symptom after *C. shiraiana* infection. CK: mulberry plants treated with empty vectors were used as controls; MaSPL8-T1 and T2: independent mulberry plants with downregulation of *MaSPL8* by VIGS treatment. Data are presented as means ± SD of three biological replicates. The significance was marked using ** (0.001 < *p* < 0.01), **** (*p* < 0.0001).

Virus-induced gene silencing (VIGS) effectively downregulated *MaSPL8* expression, with suppression detectable 9 days post-treatment and sustained for over 19 days (Figure 3A). Fourteen mulberry plants with downregulation of *MaSPL8* to varying degrees were finally obtained and were used for the following experiments (Figure S1). *MaSPL8*-silenced lines exhibited pronounced growth retardation compared to empty vector controls (Figure 3B). Notably, silenced plants displayed accelerated cell death upon *C. shiraiana* challenge, indicating susceptibility to *C. shiraiana* infection (Figure 3C,D).

### 2.4. MaSPL8 Silencing Confers Drought and Salt Stress Tolerance

**Figure 4:**
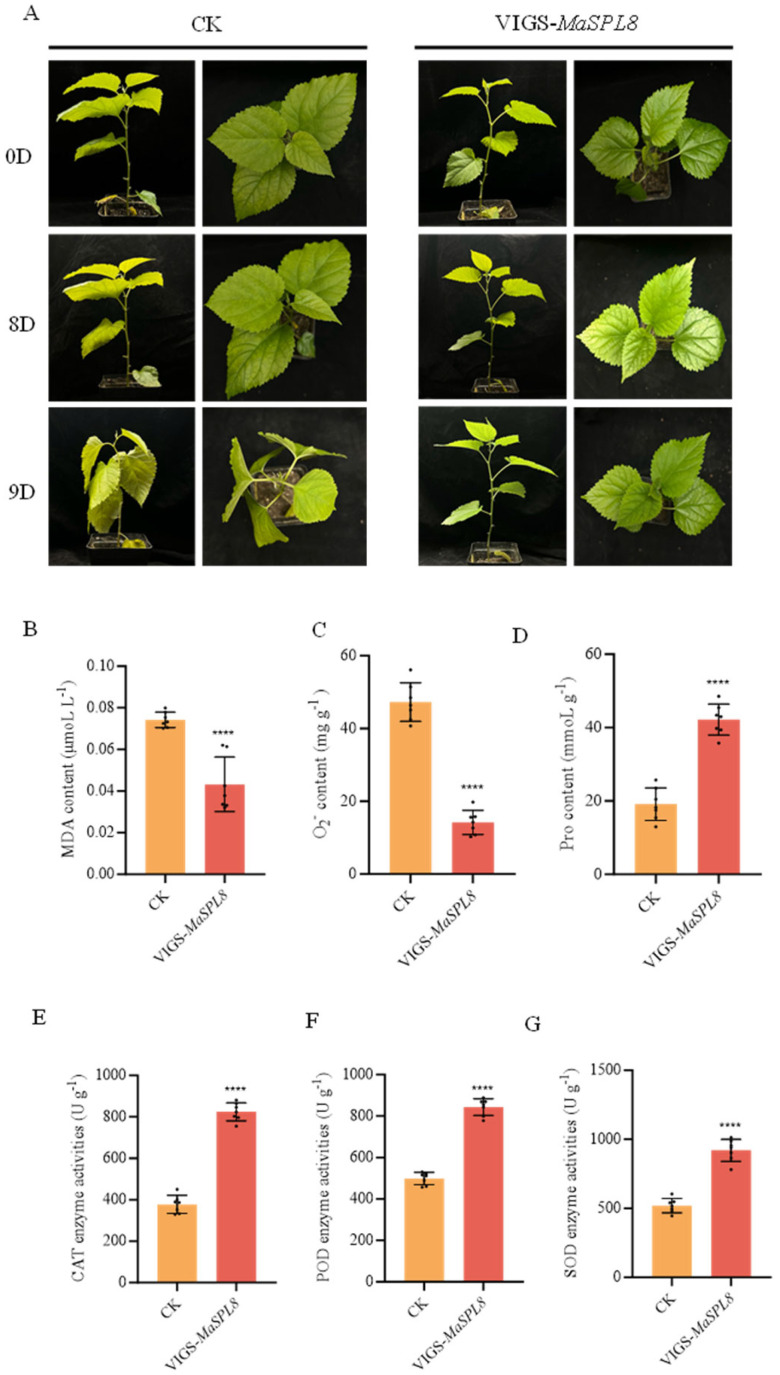
Downregulation of *MaSPL8* by VIGS increases drought tolerance in mulberry. (**A**) Growth conditions of VIGS-treated mulberry and controls under drought stress; plants were observed and recorded until the nineth day after being exposed to drought stress. (**B**) MDA contents in VIGS-treated mulberry and controls after being exposed to drought stress. (**C**) O_2_^•−^ contents in VIGS-treated mulberry and controls after being exposed to drought stress. (**D**) Proline contents in VIGS-treated mulberry and controls after being exposed to drought stress. (**E**) CAT activities in VIGS-treated mulberry and controls after being exposed to drought stress. (**F**) POD activities in VIGS-treated mulberry and controls after being exposed to drought stress. (**G**) SOD activities in VIGS-treated mulberry and controls after being exposed to drought stress. Physiological indicators were determined in leaves on the nineth day of stress treatment. CK: mulberry plants treated with empty vectors were used as controls. Three independent mulberry plants with downregulation of *MaSPL8* by VIGS treatment were used. Data are presented as means ± SD of three biological replicates. The significance was marked using **** (*p* < 0.0001).

**Figure 5:**
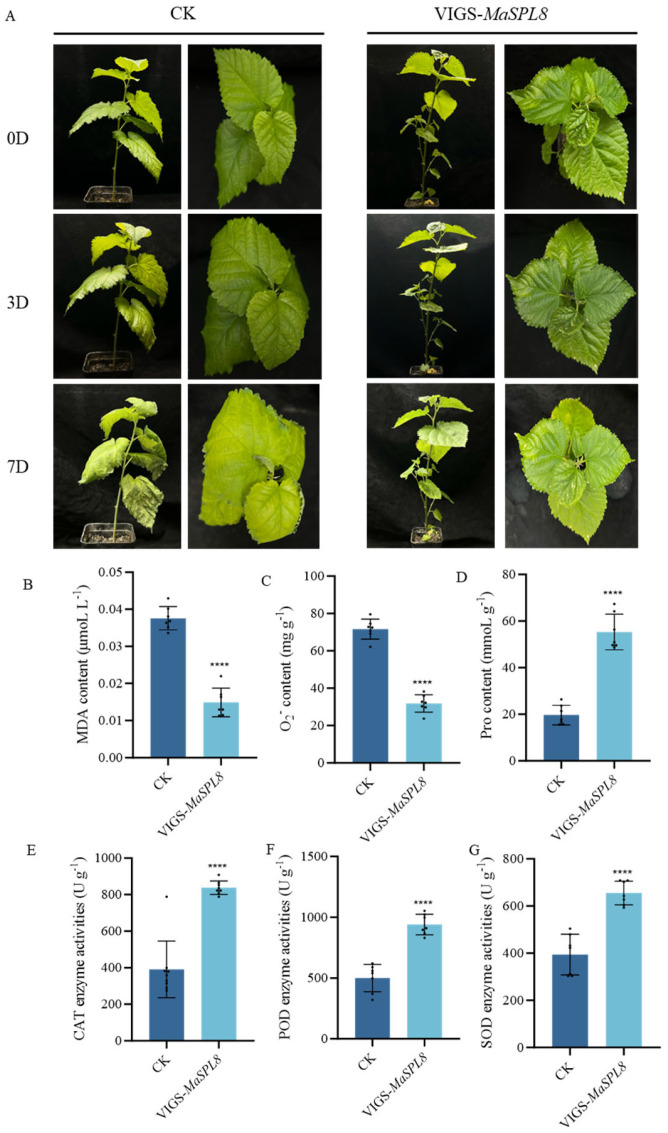
Downregulation of *MaSPL8* by VIGS increases salt tolerance in mulberry. (**A**) Growth conditions of VIGS-treated mulberry and controls under salt stress; plants were observed and recorded until the seventh day after being exposed to drought stress. (**B**) MDA content in VIGS-treated mulberry and controls after salt stress exposure. (**C**) O_2_•^−^ content in VIGS-treated mulberry and controls after salt stress exposure. (**D**) Proline contents in VIGS-treated mulberry and controls after being exposed to salt stress. (**E**) CAT activities in VIGS-treated mulberry and controls after being exposed to salt stress. (**F**) POD activities in VIGS-treated mulberry and controls after being exposed to salt stress. (**G**) SOD activities in VIGS-treated mulberry and controls after being exposed to salt stress. Physiological indicators were determined in leaves on the seventh day of stress treatment. CK: mulberry plants treated with empty vectors were used as controls. Three independent mulberry plants with downregulation of *MaSPL8* by VIGS treatment were used. Data are presented as means ± SD of three biological replicates. The significance was marked using **** (*p* < 0.0001).

Quantitative real-time PCR (qRT-PCR) confirmed the downregulation of *MaSPL8* in VIGS-treated seedlings (Figure S1). The mulberry seedlings were then divided into groups for subsequent treatments, each consisting of three VIGS lines and corresponding controls (CKs). By day 9 of drought stress, control plants showed severe leaf wilting, whereas the silenced lines retained turgid leaves (Figure 4A). Physiological assays revealed that silenced plants accumulated 42% less malondialdehyde (MDA) and 70% less superoxide anion (O_2_^•−^), alongside about 1.2-fold higher proline levels and elevated activities of SOD (EC 1.15.1.1), POD (EC 1.11.1.7), and CAT (EC 1.11.1.6) (Figure 4B–G). Similarly, under salt stress, silenced plants showed delayed leaf wilting and analogous improvements in oxidative stress markers were determined (Figure 5). These findings collectively demonstrate that *MaSPL8* acts as a negative regulator of abiotic stress tolerance.

### 2.5. Transcriptomic Analysis Reveals the MaSPL8-Regulated Pathways

**Figure 6:**
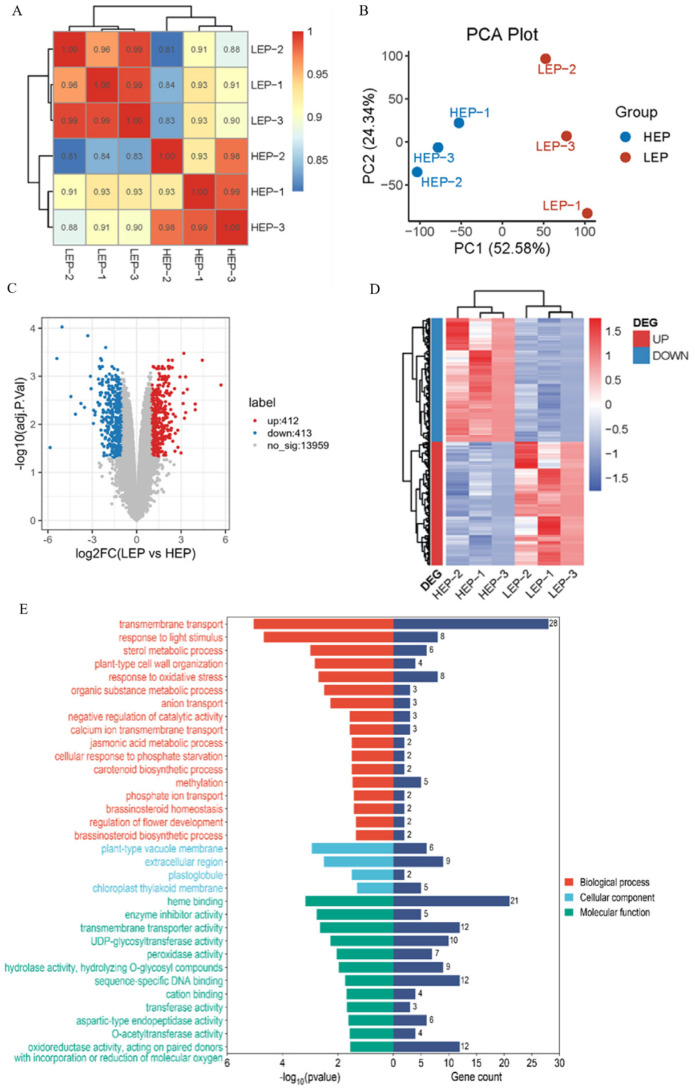
Comparative transcriptome analysis of mulberry plants with different *MaSPL8* expression levels. (**A**) Correlation of RNA-seq data from mulberry plants with different *MaSPL8* expression levels. HEP: high expression level of *MaSPL8*; LEP: low expression level of *MaSPL8*; (**B**) PCA of different samples; (**C**) volcano diagram of DEGs; (**D**) cluster and heatmap of DEGs; (**E**) GO enrichment of DEGs.

Mulberry plants with different *MaSPL8* expression levels were obtained using VIGS treatments. Two groups of mulberry plants with relatively high expression level (HEP) and low expression level (LEP) of *MaSPL8* were selected for RNA-seq (Figure S1). Correlation analysis of all samples based on RNA-seq data showed that the different groups were well distinguished, and the samples in the same group showed high correlation (Figure 6A). Principal component analysis (PCA) confirmed clear separation between HEP and LEP groups, indicating that differences mainly resulted from the change in *MaSPL8* expression levels (Figure 6B). RNA-seq analysis of *MaSPL8* HEP and LEP groups revealed 825 differentially expressed genes (DEGs: 412 upregulated, 413 downregulated) (Figure 6C,D). GO enrichment highlighted DEG associations with light signaling, oxidative stress response, floral development, and phytohormone biosynthesis (Figure 6E and Figure S2). It is notable that plant hormone biosynthesis-related processes, including sterol biosynthesis, carotenoid biosynthesis, brassinosteriod biosynthesis, and homeostasis, and the jasmonic acid metabolic process were significantly enriched, implying the important role of *MaSPL8* in regulating brassinosteriod or jasmonic acid homeostasis (Figure 6E and Figure S2).

## 3. Discussion

The functional characterization of *MaSPL8* in mulberry reveals its different roles in biotic and abiotic stress responses. Although *SPL* genes are widely implicated in stress adaptation, their functional dichotomy across stress types remains understudied. In pineapple, SBP family genes were proven to respond to abiotic stresses such as cold, heat, and salt and drought stresses. Nine of thirty *SPL* genes in poplar have been identified as candidate regulators of resistance to high salt stress [13]. The *miRNA156/157*-SPL module regulates plant resistance to various stresses in plants [37]. For instance, it was reported that *miR156* improved drought resistance of alfalfa by silencing SPL13, indicating that *SPL13* played a negative role in abiotic stress responses [38]. Non-miRNA156-targeted *SPL8* negatively regulated plant resistance to drought and salt stresses.

Downregulation of *SPL8* improved both the biomass yield and the salt/drought tolerance of transgenic alfalfa [27]. *CpSPL8* inhibited the SOS pathway and negatively regulated salt tolerance in *Codonopsis pilosula* [28]. The downregulation of *MaSPL8* in this study led to reduced malondialdehyde (MDA) and superoxide anion levels, alongside elevated proline content and antioxidant enzyme activities (SOD, POD, CAT), aligning with the classic hallmarks of enhanced stress tolerance. These physiological changes likely stem from *MaSPL8’s* transcriptional regulation of genes involved in oxidative stress mitigation and osmotic adjustment. Contrasting its abiotic stress role, MaSPL8 positively regulates biotic stress resistance. Silenced plants exhibited accelerated cell death upon *C. shiraiana* infection, a phenotype of hyper-sensitive responses (HRs) in pathogen defense. This corresponds with reports in grape where VpSBP5 activates salicylic acid (SA) or jasmonic acid (JA) signaling to the defense of Erysiphenecator [39] and mulberry *MnSPL7*, which enhances catechin biosynthesis against silkworm herbivory [35].

Transcriptomic profiling revealed that *MaSPL8* is intricately involved in the crosstalk between brassinosteroid (BR) and jasmonic acid (JA) signaling pathways. Specifically, differentially expressed genes (DEGs) associated with BR biosynthesis (including sterols and carotenoids) and JA metabolism were significantly enriched. In plants, BRs typically promote growth and enhance stress tolerance [40,41]. This supports the hypothesis that *MaSPL8* integrates BR signaling in stress adaptation, a role that parallels the function of *AtSPL8* in *Arabidopsis*, where it regulates BR signaling during anther development [29]. Additionally, SA and JA signaling pathways play critical roles in plant defense. In mulberry fruit infected with *C. carunculoide*, SA signaling is activated while JA signaling is inhibited, highlighting the complex interplay between these hormones in pathogen defense [42]. This suggests that *MaSPL8* might function in conjunction with the JA pathway to enhance resistance to *C. shiraiana* infection. The precise molecular mechanisms underlying the interplay between MaSPL8 and these hormone pathways require further investigation, including hormone profiling and promoter-binding assays to confirm the regulatory roles of *MaSPL8*.

Beyond hormone signaling, transcriptomic analysis revealed that *MaSPL8* is involved in the regulation of other pathways. For instance, 28 DEGs associated with transmembrane transport were identified, suggesting that *MaSPL8* may influence cellular transport processes during stress responses. Transmembrane transporters, such as aquaporins and cation/H^+^ exchangers, are crucial for maintaining ion homeostasis and osmotic balance under stress conditions [43]. Further exploration of these transporters could provide insights into how *MaSPL8* modulates stress tolerance at the cellular level. Moreover, the differential expression of stress-responsive transcription factors, such as members of the WRKY and MYB families, was observed. These transcription factors are known to play pivotal roles in oxidative stress tolerance, further underscoring the multifaceted regulatory network governed by *MaSPL8*.

The identification of *miR5658* and *miR4221* as potential regulators of MaSPL8 introduces an additional layer of post-transcriptional complexity to its regulation. *miR156/157* are well-known core regulators that target most SPLs and influence various biological processes. Unlike Arabidopsis SPL8, which lacks *miRNA156/157* binding sites, mulberry MaSPL8 may be modulated by novel miRNAs, reflecting lineage-specific regulatory adaptations. In fact, besides *miR156*, *miR529* has been reported to cooperate with *miR156* to target SPLs such as ZmSPL6, affecting inflorescence development [19]. In addition, *miR529* also participates in auxin-mediated phyllody in the witches’ broom disease of jujube and response to drought stress in maize [20,44]. The novel *miR5658* and *miR4221* were predicted to target MaSPL8 and studies on these two microRNAs are limited. *miR4221* was predicted to respond to cold stress in *Solanum aculeatissimum* [45] and *miR5658* participates in the regulation of internode elongation of sugarcane [46]. The functions of *miR5658* and *miR4221* in mulberry and most other plants are undetermined. The findings in the present study highlight the need to dissect miRNA-SPL networks in non-model plants, where regulatory plasticity drives ecological adaptation.

## 4. Materials and Methods

### 4.1. Plant Materials

The materials for this study were sourced from the National Mulberry GeneBank (NMGB) in Zhenjiang (32°11′ N, 119°27′ E), Jiangsu Province, China. Leaves, buds, stems, and roots of one-year-old *M. alba*-variety *Fengchi* were collected for molecular cloning and tissue expression profiling. Drought, waterlogging, low-temperature (4 °C), and high-temperature (40 °C) treatments were conducted as described previously [40,41], with leaves collected for expression analysis. High salt treatment followed our established protocol, using 200 mM NaCl irrigation until wilted, dark leaves with yellowing margins appeared. Mulberry leaves were harvested to assess *MaSPL* expression changes under various stressors. *M. alba* var. *Fengchi* seedlings were germinated in moist dishes, transplanted into pots, and grown in a growth chamber at 22 °C with a 16/8 h day/night cycle and 40–60% humidity. Virus-induced gene silencing (VIGS) was performed on four-euphylla-stage seedlings, as previously reported [42]. VIGS-treated seedlings with significant *MaSPL8* downregulation compared to controls were exposed to drought or salt stress. Seedlings with varying *MaSPL8* expression levels were used for RNA-seq to identify co-expressed genes. All samples were flash-frozen in liquid nitrogen and stored at −80 °C until analysis. Each experiment included at least three biological replicates.

### 4.2. Cloning and Characterization of MaSPL8 in Morus

Isolation of total RNA and cDNA synthesis were performed according to our previous reports. Primers used for cloning *MaSPL8* were designed according to the sequence from *M. alba* that has been reported in our previous study [40,41]. *MaSPL8* was cloned via three-step PCR at a gradient annealing temperature of 52 °C. The PCR products were purified using SanPrep column DNA gel recovery kit (Sangon Biotech, Shanghai, China) and confirmed by Sanger sequencing. The validated sequences were deposited in GenBank with accession number PV138130. The putative MaSPL8 protein sequence was aligned with other SPL homologs from *Arabidopsis thaliana* and orthologs from different species using the DNAman 8.0 software (Lynnon BioSoft, https://dnaman.software.informer.com/8.0/, accessed on 22 March 2023) with default parameters. SPB domain including Zn1 motif, Zn2 motif, and NLS was identified and marked in the alignment and logo diagram. MEME suit (http://meme-suite.org/tools/meme, accessed on 20 March 2023) was used to scan the conserved motifs of SPL8s and the logo diagram was obtained by visualizing the alignment of SPB domains using Tbtools V 2.150. A neighbor-joining (NJ) phylogenetic tree was constructed using MaSPL8 protein sequence and SPLs from *Arabidopsis thaliana*, using MEGA11.0 with JTT + G model and bootstrap test with 1000 replicates [38,39]. MicroRNA sequences from *M. alba* were extracted according to the annotation file. Both the microRNA sequences and MaSPL8 were submitted to psRNATarget (https://www.zhaolab.org/psRNATarget/analysis?function=3, accessed on 22 March 2023) to search the possible MiRNA targeting on MaSPL8.

### 4.3. Expression Profiles of MaSPL8

*MaSPL8* expression levels in various organs and under different stress conditions were analyzed by qRT-PCR using the ABI StepOnePlus™ Real-Time PCR System (ABI Applied Biosystems, Waltham, MA, USA). The reaction mix included 2× ChamQ™ SYBR^®^ qPCR Master Mix (Vazyme, Nanjing, China) and 50× ROX Reference Dye 1. The qRT-PCR protocol involved a two-step cycling program: 95 °C for 2 min, followed by 40 cycles of 95 °C for 10 s and 60 °C for 30 s, with *Actin* as the reference gene. Results were visualized using GraphPad Prism 8.0 and ANOVA was performed with *p* < 0.05 considered significant. Each experiment included three biological or technical replicates.

### 4.4. Obtaining Mulberry with DownRegulated MaSPL8 Using VIGS

We used virus-induced gene silencing (VIGS) to generate mulberry trees with varying levels of *MaSPL8* downregulation [43]. Recombinant plasmids for VIGS were constructed using Nimble cloning [44] and VIGS was performed as described previously [41,42,43]. Mulberry leaves were infected via pressure injection, with empty vectors pTRV2 and pTRV1 as negative controls. *MaSPL8* expression levels were measured by qRT-PCR 9 and 19 days post-injection. Knockdown efficiency was calculated by comparing *MaSPL8* expression in VIGS-treated plants to controls.

### 4.5. Estimation of Plant Resistance to C. shiraiana Infection

Cell death symptoms and *C. shiraiana* growth were recorded to evaluate transgenic plant resistance to infection, as previously described [41]. *C. shiraiana* was inoculated 10 days after infiltration in mulberry. Results are based on at least three biological replicates.

### 4.6. Drought and Salt Stress Treatment and Physiology Indicator Determination

Mulberry plants with confirmed *MaSPL8* downregulation via VIGS were subjected to drought or salt stress. For drought stress, plants were placed in a single pot without irrigation, while controls received regular watering. For salt stress, plants were irrigated daily with 200 mM NaCl instead of water. Drought stress lasted seven days and salt stress continued for nine days. Plant growth conditions were photographed to document tolerance to drought or salt stress. Physiological indicators, including MDA and proline contents, O_2_^−^ levels, and SOD, POD, and CAT activities in leaves, were measured using samples collected on the seventh or ninth day, as described previously [45,46]. Data were analyzed using GraphPad Prism 8.0, with ANOVA and visualization performed at a significance level of *p* < 0.05. All experiments included three biological replicates.

### 4.7. RNA-Seq and Comparative RNA-Seq Analysis

The trimmed and filtered reads were aligned to the *M. alba* genome released by Jiao et al. (2020) [47] using bowtie2 (version-2.3.2) [48]. Samtools was used to operate the bam files. StringTie v2.15 was used to calculate the expression matrix with the genome annotation file (.gff3) [49]. Tbtools V 2.150 was used to identify differentially expressed genes and perform GO enrichment analysis [50]. R version 4.1.2 was used for R-package-based analyses.

## 5. Conclusions

In summary, this study establishes *MaSPL8* as a multifaceted regulator in mulberry, positively influencing resistance to fungal pathogens and negatively influencing drought resistance and salt stress. Its downregulation enhances abiotic stress tolerance by bolstering antioxidant capacity and osmotic regulation, while concurrently intersecting with BR and JA signaling pathways. The species-specific functional divergence of SPL8 homologs highlights the evolutionary plasticity of this transcription factor family. Our work not only expands upon the functional repertoire of *SPL8* genes but also provides a genetic target for improving mulberry’s stress resilience—a critical trait for its roles in sericulture and phytoremediation. Future studies should prioritize elucidating the molecular mechanisms underlying MaSPL8 hormone crosstalk and validating the roles of *miR5658/miR4221* in its regulation.

## Figures and Tables

**Table 1:** Prediction of upstream miRNA targeting on *MaSPL8*.

miRNA	Target Gene	Expect	Alignment	Inhibition
*miR5658*	*MaSPL8*	2.5	miRNA 21 AAAGUAGUAGUAGUAGUAGUA 1	Cleavage
			:: :::::::::: :::::	
			Target 154 ACUCCUCAUCAUCAUAAUCAU 174	
*miR4221*	*MaSPL8*	5	miRNA 22 CGUUCUUAAGUUGUCUCCUUUU 1	Cleavage
			.::.: ::.:.::::...:	
			Target 417 CGGAGGAGGCAGCGGAGGGGGA 438	

## Data Availability

Data is contained within the article or Supplementary Materials.

## Supplementary Materials

The following supporting information can be downloaded at https://www.mdpi.com/article/10.3390/plants14060950/s1: Figure S1: Expression levels of *MaSPL8* in VIGS-treated mulberry. Figure S2: GO enrichment analysis of DEGs. (A) GO enrichment of up-regulated DEGs; (B) GO enrichment of downregulated DEGs.
