# Supplementary material for: Functional Characterization of MaSPL8 Reveals Its Different Roles in Biotic and Abiotic Stress Responses in Mulberry
Source: Plants (Basel). 2025 Mar 18;14(6):950. doi: 10.3390/plants14060950 (PMC11944325; doi:10.3390/plants14060950)
Supplement: Supplementary file 1 [file plants-14-00950-s001.zip › plants-3514812-supplementary.pdf]

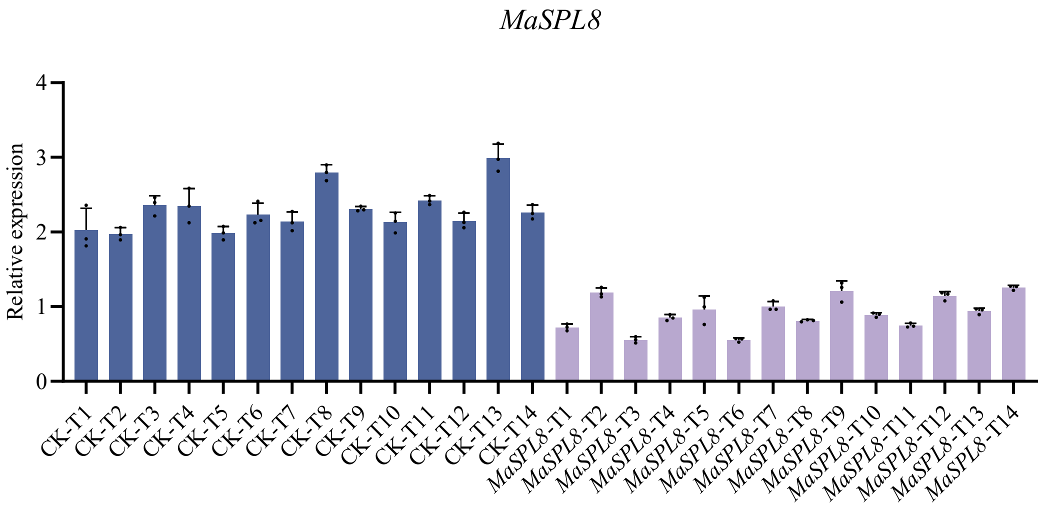

Figure S1 Expression levels of *MaSPL8* in VIGS treated mulberry. CK: mulberry plants treated with empty vectors were used as controls; MaSPL8-T1 -T14: Independent mulberry plants with down-regulation of MaSPL8 by VIGS treatment. Data are presented as means ± SD of three biological replicates. The significance was marked using*$(0.01<p<0.05)$, **$(0.001<p<0.01)$, ****$(p<0.0001)$.

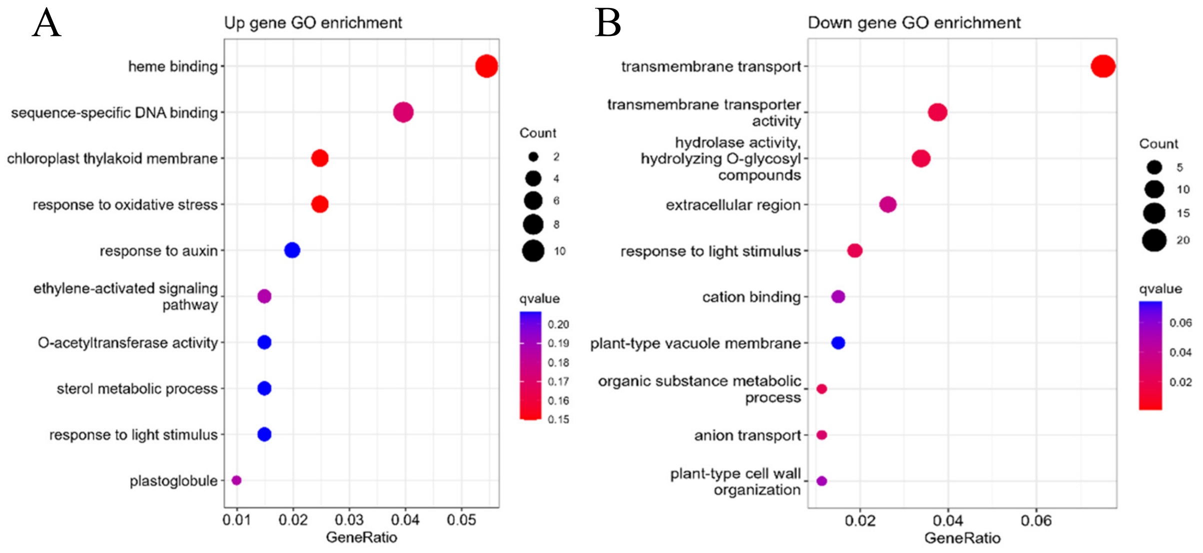

Figure S2 GO enrichment analysis of DEGs. A: GO enrichment of up-regulated DEGs; B: GO enrichment of down-regulated DEGs.
